# Novel Consensus Splice Site Pathogenic Variation in THOC2 Gene Leads to Recurrent Arthrogryposis Multiplex Congenita Phenotype: A Case Report

**DOI:** 10.7759/cureus.19682

**Published:** 2021-11-17

**Authors:** Vasundhara Tamhankar, Parag Tamhankar, Rajas Chaubal, Jyoti Chaubal, Nitin Chaubal

**Affiliations:** 1 Genetics, Centre for Medical Genetics, Mumbai, IND; 2 Genetics, MedGenome Labs, Bengaluru, IND; 3 Radiology, Thane Ultrasound Centre, Thane, IND

**Keywords:** x linked recessive, mrna export, arthrogryposis multiplex congenita, splice, mutation, thoc2

## Abstract

The *THOC2* gene encodes THO complex subunit 2, a subunit of the Transcription-Export (TREX) complex which binds specifically to splice messenger ribonucleic acid (mRNAs) to facilitate mRNA export. Mutations in the *THOC2* gene have been described to lead to X-linked mental retardation syndrome type 12/35 (XLMR-12/35) (MIM#300957). Here, we describe for the first time a recurrent arthrogryposis multiplex congenita phenotype (AMC) in two male fetuses in a family. Exome sequencing identified a novel pathogenic variation chrX: 122761817_122761820delTGAC (genome assembly GRCh37 format) or c.2482-1_2484delGTCA (as per Genbank transcript ID NM_001081550) in the*THOC2* gene. This variant affects the consensus acceptor splice site between intron 22 and exon 23. This is the most severe phenotype described in *THOC2* gene-related disease till date. This case report expands the clinical phenotype of *THOC2* gene related defects.

## Introduction

The transcription/export (TREX) multiprotein complex facilitates the transport of spliced messenger ribonucleic acid (mRNAs) out of the nucleus into the cytoplasm for further essential processes in neuronal development. It is ubiquitously expressed and most importantly in the developing and mature frontal cortex and cerebellum [1]. The THO complex subunit 2 encoded by the* THOC2* gene on chromosome locus Xq25 forms an important subunit of the TREX complex along with proteins ALY (encoded by *THOC4 *gene) and UAP56 (encoded by gene *DDX39B*). Pathogenic variants in the *THOC2* gene have been known to lead to X-linked mental retardation (XLMR) referred to as XLMR type 12 and XLMR type 35 (MIM#300957) [2,3]. Clinical features known in this syndrome are variable intellectual disability, microcephaly, variable facial dysmorphism, speech delay, short stature, obesity, hypotonia, tremor, gait disturbance, behavioral abnormalities, seizure disorder, brain ventriculomegaly, gliosis, inferior cerebellar vermis dysplasia, and cervical cord compression. Pathogenic variants reported in these patients with XLMR have been of missense type or splice site pathogenic variants which have led to truncated protein/unstable protein formation with partial loss of activity. The *THOC2* gene pathogenic variants with null protein activity are postulated to be lethal in fetal life but have not been observed in medical literature. In this study, we describe the clinical and genetic findings of a novel severe fetal phenotype namely arthrogryposis multiplex congenita in two male fetuses of a couple.

## Case presentation

**Figure 1:**
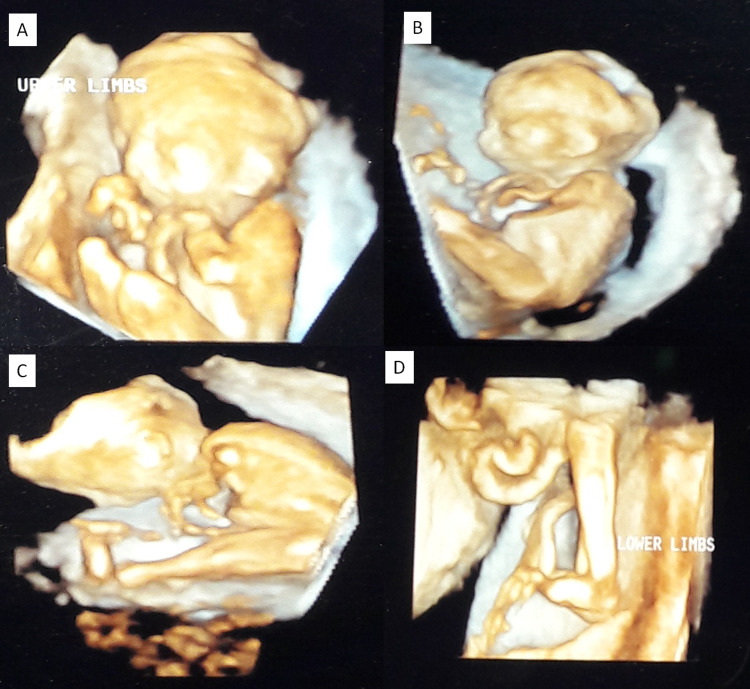
Antenatal ultrasound in the first pregnancy 3D rendered images of the antenatal ultrasound show the fetus having arthrogryposis multiplex congenita (AMC) features: bilateral persistent clenched fists (A), flexion at the shoulders and elbows (B), flexion at hip and hyperextension at the knees (C), club feet (D).

A non-consanguineous couple of Indian ethnicity and belonging to the Hindu Brahmin community from the state of Maharashtra, India presented in their first pregnancy with multiple anomalies detected at around 19 weeks of gestation. The ultrasound showed a biparietal diameter of 4.5 cm (mean for 19 weeks and six days), head circumference of 17.10 cm (mean for 19 weeks and five days), abdominal circumference of 13.14 cm (mean for 19 weeks), femur length of 3.10 cm (mean for 19 weeks and six days). Both lower limbs were hyperextended at the knee joints and bilateral rocker bottom feet were present. Both upper limbs were persistently flexed at the elbow and wrist joints. Both hands showed persistently clenched fists (see Figure 1 A to D). Generalized subcutaneous edema was noted. A small stomach bubble was seen. The nuchal fold was 6 mm (prominent). Transcerebellar diameter was 20.7 mm (normal), cisterna magna was 4.1 mm (normal), lateral cerebral ventricles measured 6.9 mm (normal). The spine, heart four-chamber views, kidneys, urinary bladder, diaphragm, and bowels were normal. The possibility of arthrogryposis multiplex congenita (AMC) was considered and parents opted for medical termination of pregnancy.

**Figure 2:**
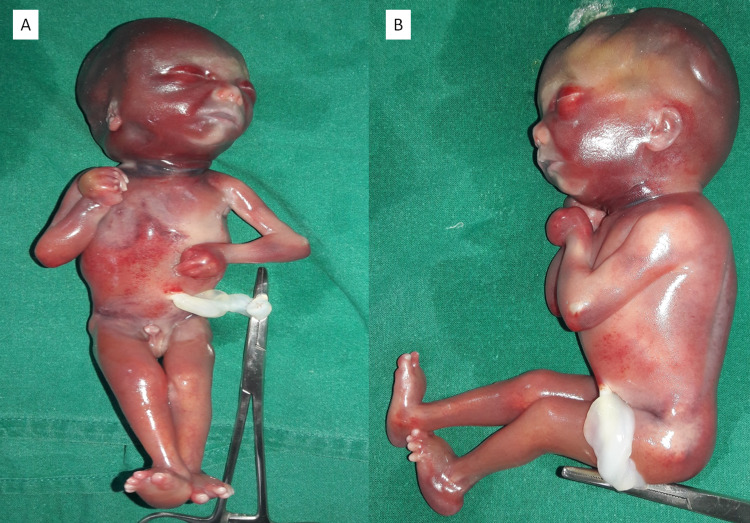
Autopsy images of the fetus from the first pregnancy A: Frontal view, B: Lateral view shows dysmorphic facial features, generalized subcutaneous edema and AMC AMC: Arthrogryposis multiplex congenita

A postnatal autopsy was conducted on the fetus (see Figure 2). The anthropometry was as follows: fetal weight of 450 gms (mean for 22 weeks), total length 22 cm (mean for 17 - 18 weeks), crown-rump length 16 cm (mean for 19 - 20 weeks), arm 4.5 cm, forearm 3.5 cm, thigh 5 cm, leg 4 cm, and foot 3.5 cm (mean for 20 weeks), head circumference was 17 cm (mean for 20 weeks). The baby had generalized subcutaneous edema. The face was round in shape with a short columella, upturned nares, flat nasal bridge, pursed lips, and micrognathia. There was no cleft lip/palate. The skull, spine, thorax, and abdomen were normal on external examination. Arthrogryposis multiplex congenita findings were noted. The elbows were fixed in flexion and hands were clenched. Knees were persistently hyperextended. There was bilateral rocker bottom foot. The umbilical cord had three vessels, two arteries, and one vein. The external genitalia was that of a male, testes were in the abdomen. The anal opening was patent. Internal examination of the abdomen and thorax revealed normal findings. There were neither ascites nor pleural/pericardial effusion. The diaphragm was intact. There was no liver or spleen enlargement. Testes and epididymal structures were noted bilaterally. The stomach and the intestinal tract were normal. The kidneys did not show any cysts. The heart was normal. The pulmonary trunk and the aorta opened into the right and left ventricle, respectively. No ventricular septal defect could be noted. The atrioventricular openings were normal. The pulmonary veins drained into the left atrium. The lungs were normal in size and did not show any cysts on the cut surface. The thymus was normal. There was no evidence of trachea-esophageal fistula. The brain showed normal structure on internal examination. Complete fetal radiographs did not show any signs of any specific skeletal dysplasia.

**Figure 3:**
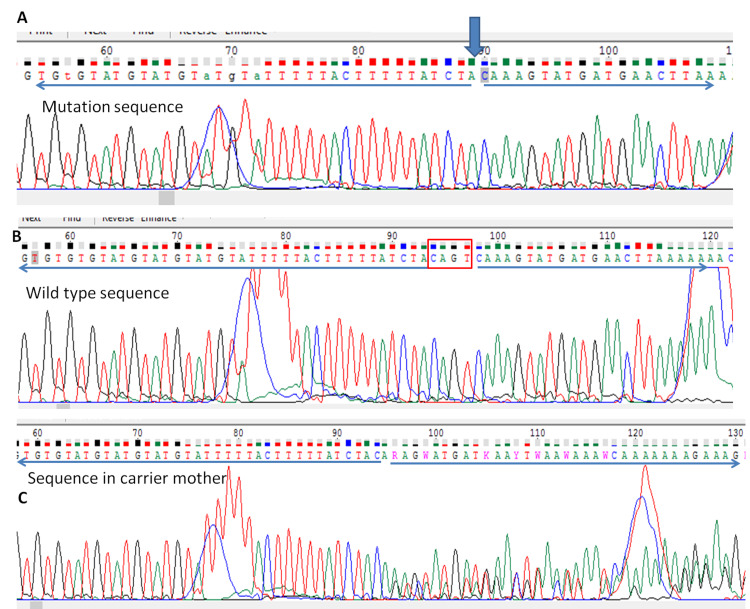
Sequence chromatograms of THOC2 gene Sequence chromatograms of the mutant allele show hemizygous deletion of the four nucleotides - adenine, cytosine, guanine, and thymine (ACGT). A shows the deletion position indicated by the blue arrow. B indicates wild type/normal allele. C reveals heterozygous deletion in carrier mother.

On a genetic study of the first fetus, the chromosomal microarray (Affymetrix platform, Optima chip containing 315K probes) did not reveal any significant chromosomal aneuploidies or deletion/duplication syndromes. The clinical exome (6500 clinically relevant genes) was captured using custom-designed Agilent Sureselect (Agilent, CA, USA) to target specific probes. These targeted regions were then sequenced using the Illumina sequencing system (Illumina Inc., CA, USA) at a mean coverage of more than 80-100X, and read quality more than Q20. The target region included the exon and 10bp of flanking intronic sequence. Bioinformatic analysis from variant call format (VCF) file and interpretation of sequence variants was done. The bioinformatics pipeline used for analyzing/annotation of VCF files was Annovar (http://wannovar.wglab.org/). Variants were considered pathogenic if they were previously reported in Online Mendelian Inheritance in Man (OMIM) indexed disorders or, if they are pathogenic as per American College of Medical Genetics (ACMG) criteria [4]. Variants were also considered pathogenic if previously reported in the ClinVar database and thought to have important clinical implications. Low somatic mosaic novel variants for dominant diseases with variable penetrance were not considered significant. Low quality and low coverage data were filtered out. Also, the type of mutation identified had to have a known biological mechanism for the linked disease as per scientific publications or guidelines. Also, the variant should not have been reported in a homozygous state in Exome Aggregation Consortium (ExAC) databases. No pathogenic mutations were detected in the known genes for AMC namely *ACTA1, AGRN, ALG14, ALG2, ALG3, ANTXR2, BICD2, BIN1, CHAT, CHMP1A, CHRNA1, CHRNB1, CHRND, CHRNE, CHRNG, CHST14, CNTN1, CNTNAP1, COL6A1, COL6A2, COL6A3, COLQ, DHCR24, DNM2, DOK7, DPAGT1, ECEL1, EGR2, ERBB3, ERCC1, ERCC5, ERCC6, EXOSC3, FBN1, FBN2, FKBP10, FKRP, FKTN, FLVCR2, GBA, GBE1, GFPT1, GLE1, GMPPB, IGHMBP2, KAT6B, KLHL40, KLHL41, LMNA, LMOD3, LRP4, MAGEL2, MPZ, MTM1, MUSK, MYBPC1, MYH2, MYH3, MYH8, NALCN, NEB, PIEZO2, PIP5K1C, PLOD2, RAPSN, RIPK4, RYR1, SCARF2, SCN4A, SELENON, SKI, SLC35A3, SLC39A13, SLC5A7, SOX10, SYNE1, SYT2, TGFB3, TNNI2, TNNT1, TNNT3, TPM2, TPM3, TRIP4, TRPV4, TSEN54, TTN, UBA1, VIPAS39, VPS33B, ZC4H2*. The fetus was hemizygous for deletion of four nucleotides at the acceptor splice site of intron 22 and exon 23 boundary, namely chrX: 122761817_ 122761820delTGAC (as per genome assembly GRCh37 or hg19 format), or c.2482-1_2484deletionGTCA( as per Genbank transcript ID NM_001081550 with coverage at 39x depth). This was confirmed on Sanger sequencing in the fetus, and the mother was the carrier for the same mutation (see Figure 3).

**Figure 4:**
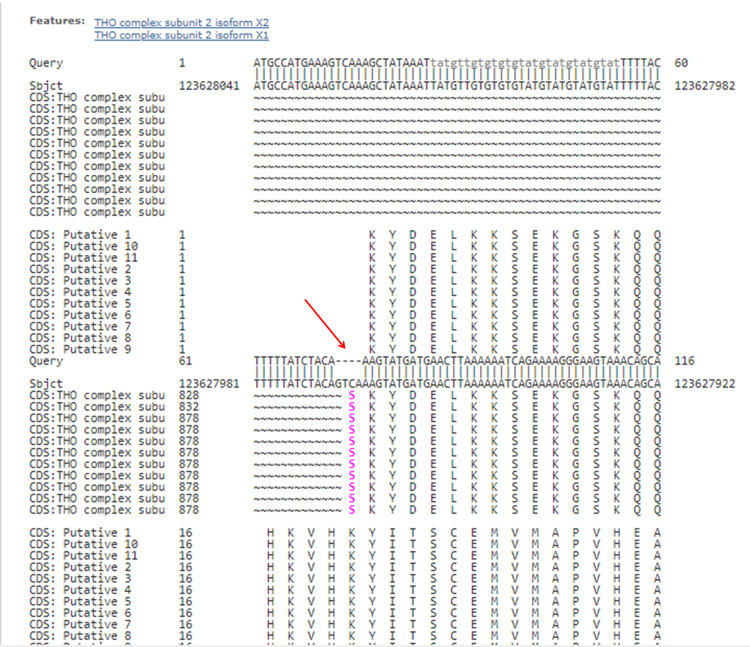
BLAST analysis of the THOC2 gene mutation Basic Local Alignment Search Tool (BLAST) showing deletion of the four nucleotides - adenine, cytosine, guanine, and thymine (ACGT) - at the acceptor splice site between intron 22 and exon 23 (indicated by red arrow).

This mutation affects the canonical +/- one or two splice sites as seen from the Basic Local Alignment Search Tool (BLAST) analysis (see Figure 4). The pathogenic variant was also examined by the Variety Seeking Tendency Scale (VARSEEK). It showed that a class five deleterious effect (score +39.28 %) on the acceptor splice site is produced by this variant, with the likely mechanism being exonic skipping. Exome sequencing (panel including 6500 clinically relevant genes) in parents was also carried out, which confirmed the mother to be the carrier for the same mutation. No other clinically relevant mutation was present in the couple. The parents were examined to be clinically normal

**Figure 5:**
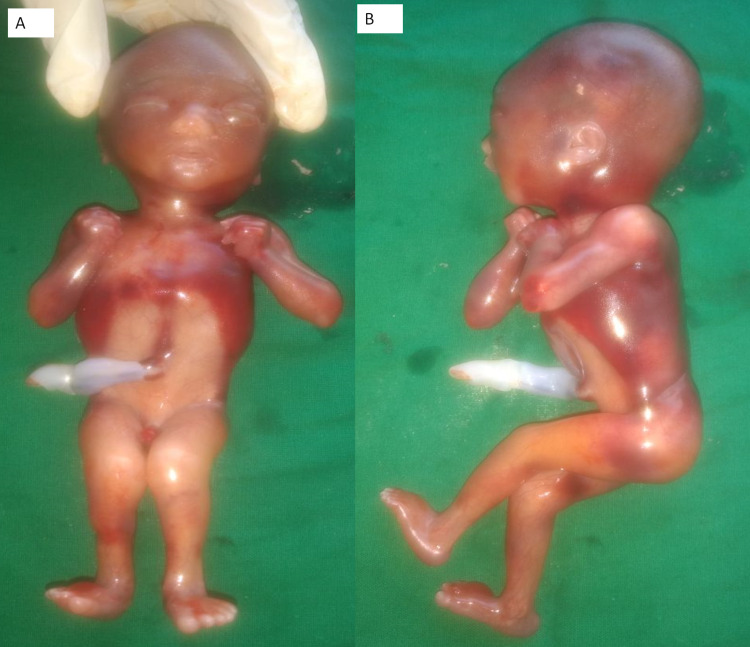
Autopsy images of the fetus from the second pregnancy A: Frontal view, B: Lateral view shows dysmorphic facial features, generalized subcutaneous edema, and arthrogryposis multiplex congenita (AMC).

The couple planned a second pregnancy. Again, around 19 weeks, the fetus showed the ultrasound findings of AMC which led to parents opting for medical termination of pregnancy. This fetus too, was male. External examination and limited autopsy confirmed the findings of AMC (see Figure 5). Sanger sequencing for the variant confirmed that this fetus too, was hemizygous for the chrX: 122761817_ 122761820delTGAC mutation. Thus genetic testing confirmed that the recurrent AMC syndrome in the fetuses was due to *THOC2* gene mutation inherited in an X-linked recessive manner.

On follow-up, the family opted for the reproductive choice of in-vitro fertilization using an oocyte from an unrelated donor to avoid further recurrences and have a healthy baby as a result.

Written informed consent for publication of de-identified clinical data and images was obtained from the family.

## Discussion

Arthrogryposis multiplex congenita (AMC) is defined as the presence of non-progressive joint contractures at or before birth in more than two non-contiguous areas of the body leading to restricted joint movements [5]. It is a result of fetal akinesia or lack of fetal movement and is present as a feature in more than 400 disorders. It can be detected on antenatal ultrasound usually as abnormal joint positions and lack of observed fetal movements across or at these joints. The underlying defect is in the central nervous system, peripheral nervous system, neuromuscular junction, muscles, connective tissue, or metabolic pathways. The most common defect noted is club foot and clenched fists but larger joints such as hip, knee, elbows, shoulders can also be involved. When the cause of AMC is hypoplastic muscles, the term amyoplasia is used which accounts for 30% of all cases. Distal arthrogryposis involves only the distal muscles and has a favorable prognosis. Syndromic arthrogryposis includes Pena Shokeir syndrome (which includes restrictive dermopathy), fetal akinesia deformation sequence (intrauterine growth retardation, hydrops fetalis, cleft lip/palate, lung hypoplasia), multiple pterygium syndrome (webs of skin across a large joint), and lethal congenital contracture syndrome. Affected infants usually die in utero or very shortly after birth due to respiratory insufficiency [5]. In the present fetuses, facial dysmorphism and generalized skin edema pointed towards syndromic AMC.

We identified two male fetuses in subsequent pregnancies of a family to be affected by AMC due to a novel *THOC2* gene-splicing mutation. The present *THOC2* gene variant satisfied the ACMG criteria for pathogenicity as given in Table 1. The various mechanisms leading to the pathogenicity of splice site variants include exon skipping, shortening, or inclusion of intronic sequence due to activation of alternative/new splice site. Most are predicted to lead to a null effect but rarely, a gain of function effect may be possible if there is in-frame deletion or insertion of sequence [6]. Gain of function was not considered a mechanism for the present *THOC2* variant since the carrier mother was unaffected

**Table 1:** ACMG criteria for pathogenicity satisfied by the present variant in THOC2 gene ACMG: American College of Medical Genetics

Criteria	Type	Description	Satisfied
PVS1	Very strong	Null variant (nonsense, frameshift, canonical +/- 1 or 2 splice sites, initiation codon, single or multiexon deletion) in a gene where the loss of function is a known mechanism of disease	Yes
PP1	Strong	Co-segregation with multiple affected family members in a gene definitively known to cause disease	Yes
PM2	Moderate	Absent from controls (or at extremely low frequency if recessive) in Exome sequencing project, 1000 Genomes project or ExAC databases	Yes
PP4	Supportive	Patient phenotype of family history is highly specific for a disease with a single genetic etiology	Yes

We also reviewed published cases with *THOC2* gene mutations. There is a scarcity of papers on clinical aspects of the *THOC2* gene in medical literature. A search in PubMed using the keyword “*THOC2*” led to a list of only 17 papers. A total of three seminal papers by Kumar et al. have a clinical and genetic characterization of families with *THOC2* gene mutations [7,8,9]. The findings in these papers have been summarized in Table 2. The frequencies of important clinical features are as follows. Intellectual disability was present in all except one (only had speech apraxia). Intellectual disability was profound in 8 % (3/37 cases), severe in 29.7 % (11/37 cases), moderate in 40.5 % (16/37 cases), mild in 13.5 % (5/37 cases), and borderline in one case. Other features included speech delay in 83.7 % (31/37 cases), hypotonia in 31.4% (11/35 cases), hyperkinesia in 21.6 % (8/37 cases), tremors in 27 % (10/37 cases), epilepsy in 29.7 % (11/37 cases), gait disturbances in 54 % (20/ 37 cases), behavioral problem in 38.8 % (14/36 cases), microcephaly in 35.1 % (13/37 cases) and short stature in 54 % (20/37 cases). Mutations identified in these patients included 16 missense mutations, one exonic deletion (37, 38 exons), two splice-site mutations. The splice site mutation c.3053+4A>C led to the retention of intronic sequence between exon 28-29 predicted to result in an 1175 amino acid truncated protein containing the 1168 wild type amino acids and seven amino acids from the translated aberrant retained intronic sequence. Whereas the splice site mutation c.4450-2A>G led to the retention of the intron between exons 34 and 35 leading to a protein with 1535 amino acids (1483 amino acids from wild type *THOC2* and 52 translated from the intronic sequence) instead of the wild type 1593 amino acids. In these studies, none of the families reported a fetal phenotype or ultrasound findings of any such anomalies observed in their family. All the above mutations cause a partial loss of function of *THOC2* activity rather than a complete loss. [7,8,9] 

**Table 2:** Clinical and genetic characteristics of individuals identified with THOC2 gene-related diseases reported in medical literature aa: amino acid, acm: abnormal cerebral myelination, acv: abnormal cerebellar vermis, AP: anxiety problem, b: borderline intellectual disability, BHF: broad high forehead, BP: behaviour problems, ca: cerebellar atrophy, CCC: cervical cord compression, ch: cranial hyperostosis, CHD: congenital hip dysplasia, cvd: cerebellar vermis dysplasia, cvi: cortical visual impairment, D: depression, dm: delayed myelination, Epi: epilepsy, Fam ID: family ID, GD: gait disturbances, ghd: growth hormone deficiency, glio: gliosis, gmh: gray matter heterotopia, HT: hypotonia, HK: hyperkinesia, Idl: intellectual disability, ipf: idiopathic pulmonary fibrosis, jl: joint laxity, LBW: low birth weight, LE: large ears, lvw: increased left ventricular wall thickness on 2d echo,  lbv: low brainstem volume, LBW: low birth weight, m: moderate intellectual disability, mi :mild intellectual disability, Mic: microcephaly, mut cDNA: mutation in cDNA format, mut prot: mutation in protein change format,  N: No, nd: not documented, nf: Noonan facies, NI: neuroimaging find ngs, nr: normal, nys: nystagmus, OF: other features, OW: overweight, p: profound intellectual disability, PM: prematurity, pvm: periventricular white matter abnormality, ref: reference, rpp: right perisylvian polymicrogyria, s: severe intellectual disability, sh: subluxed hips, sm: small midbrain, stm:stereotypic movements, SS: short stature, tcc: thin corpus callosum, Tr: tremor, tw: toe walking, uls: upper limb spasticity, vgp: variability in gyral pattern, VM: cerebral ventriculomegaly, Y:Yes

Ref	Fam ID	Case ID	Age	PM	LBW	Idl	SD	HT	HK	Tr	Epi	GD	BP	AP	D	Mic	SS	OW	BHF	LE	NI	OF	Mut cDNA	Mut Prot
[7]	1	1	9	N	N	m	Y	Y	N	N	Y	Y	Y	N	N	N	N	nd	N	N	nd		c.3034T>C	p.S1012P
		2	5	N	N	b	N	Y	N	N	N	N	N	N	N	N	N	nd	N	N	nd		c.3034T>C	p.S1012P
		3	21	N	Y	m	Y	Y	N	N	Y	N	N	N	N	Y	N	nd	N	N	nd		c.3034T>C	p.S1012P
	2	4	77	Y	N	m	Y	Y	N	N	N	N	N	N	N	N	Y	N	N	Y	nd	ipf	c.1313T>C	p.L438P
		5	61	Y	N	s	N	Y	N	Y	N	Y	N	Y	N	Y	Y	Y	Y	Y	ccc, glio		c.1313T>C	p.L438P
		6	63	N	N	m	Y	N	N	Y	N	Y	Y	Y	Y	N	Y	Y	N	Y	nd		c.1313T>C	p.L438P
		7	51	N	Y	mi	Y	N	N	Y	N	N	N	N	N	Y	Y	N	N	N	nd		c.1313T>C	p.L438P
		8	51	N	N	mi	Y	N	N	Y	N	N	N	N	Y	N	Y	Y	N	N	nd	lvw	c.1313T>C	p.L438P
		9	30	Y	Y	mi	Y	Y	N	Y	N	N	N	N	N	N	Y	Y	Y	N	nr		c.1313T>C	p.L438P
		10	14	N	N	m	Y	Y	N	N	N	N	N	N	N	Y	Y	N	N	N	nd		c.1313T>C	p.L438P
		11	9	N	Y	mi	Y	Y	N	N	N	N	N	N	N	Y	Y	N	N	N	nr	ghd	c.1313T>C	p.L438P
	3	12	44	N	N	m	Y	N	Y	N	N	N	Y	N	N	N	Y	Y	N	N	nd		c.2399T>C	p.I800T
		13	43	N	N	s	Y	Y	N	N	Y	Y	Y	N	N	N	Y	Y	N	N	vm		c.2399T>C	p.I800T
		14	42	N	N	m	Y	N	N	N	N	N	N	N	N	N	Y	Y	N	N	nd		c.2399T>C	p.I800T
		15	34	N	N	m	Y	N	N	N	N	N	Y	N	N	N	Y	Y	N	N	nd		c.2399T>C	p.I800T
		16	30	N	N	m	N	N	Y	N	N	N	Y	N	N	N	N	Y	Y	N	nd		c.2399T>C	p.I800T
		17	28	N	N	m	N	N	Y	N	N	N	Y	N	N	N	Y	N	Y	N	nd		c.2399T>C	p.I800T
	4	18	24	N	N	s	Y	Y	Y	Y	Y	Y	Y	N	N	N	N	N	N	N	vm, ch	stm, uls	c.937C>T	p.L313F
		19	30	N	N	m	N	N	N	Y	Y	N	Y	N	N	N	N	Y	N	N	cvd		c.937C>T	p.L313F
		20	38	Y	N	mi	N	N	N	Y	N	N	N	N	N	N	N	N	N	N	nd	uls, chd	c.937C>T	p.L313F
[8]	5	21	12	Y	Y	s	Y	N	N	N	N	N	N	N	N	Y	Y	N	Y	nd	nr		c.2087C>T	p.T696I
	6	22	5	N	N	m	Y	Y	N	Y	Y	Y	Y	N	N	Y	Y	N	Y	nd	tcc, lbv, vgp	tw, jl, sh	c.2138G>A	p.G713D
	7	23	7	N	Y	m	Y	nd	Y	N	N	Y	Y	N	N	N	N	N	Y	nd	nd	tw, nf	c.3559C>T	p.H1187Y
	8	24	7	N	Y	m	Y	nd	Y	N	N	Y	Y	N	N	N	N	N	Y	nd	nd	nf	c.3559C>T	p.H1187Y
	9	25	3	N	N	s	Y	Y	Y	N	N	Y	Y	N	N	N	N	N	N	nd	vd, dm, pvm		c.3503+4A>C	p.G1168fs7X
	10	26	10	N	N	s	Y	Y	N	Y	N	Y	N	N	N	Y	N	N	N	nd	nr	nys	c.4450-2A>G	p.R1483fs52X
	11	27	10	nd	nd	p	Y	Y	N	N	Y	Y	nd	N	nd	N	N	N	nd	nd	nr	cvi	c.1550A>G	p.Y517C
[9]	12	28	6	N	Y	p	Y	Y	Y	N	Y	Y	N	N	N	Y	Y	N	nd	nd	dm, vm, sm		c.229C>T	p.R77C
	13	29	5	N	N	p	Y	N	N	N	N	N	N	N	N	Y	N	N	nd	nd	acm		c.1966A>G	p.N666D
	14	30	20	Y	Y	s	Y	Y	N	N	N	Y	N	N	N	Y	Y	N	nd	nd	vm		c.2170A>G	p.K724E
	15	31	5	N	N	N	Y	N	N	N	N	Y	N	N	N	N	N	N	nd	nd	nr		c.2642A>G	p.Y881C
	16	32	15	N	N	s	Y	Y	N	N	N	Y	N	N	N	N	Y	N	nd	nd	nd		c.2942G>A	p.C981Y
		33	10	N	N	s	Y	Y	N	N	N	Y	Y	N	N	N	Y	N	nd	nd	ca, pvm		c.2942G>A	p.C981Y
	17	34	2.5	N	N	m	Y	Y	N	N	Y	Y	N	N	N	Y	N	N	nd	nd	gmh		c.3223C>T	p.R1075W
	18	35	4.5	N	N	s	Y	Y	N	N	N	Y	N	N	N	N	N	N	nd	nd	dm, rpp		c.3300G>T	p.W1100C
	19	36	2	N	Y	s	Y	Y	N	N	Y	Y	N	N	N	N	Y	N	nd	nd	vm, sm, acv		c.4646A>G	p.K1549R
	20	37	11	N	N	m	Y	Y	N	N	Y	Y	N	N	N	Y	N	N	nd	nd	acm		delex37,38	C terminus 34 aa deletion

It is important to note gene *GLE1* (locus 9q34.11), which also has a similar function to the *THOC2 *gene that is nuclear mRNA export. Mutations in the *GLE1 *gene lead to an autosomal recessive syndrome namely congenital arthrogryposis with anterior horn cell disease as well as diffuse motor neuron axonopathy (CAAHD) (MIM#611890) [10]. Most patients die in infancy or childhood. Patients that die in utero are said to have lethal congenital contracture syndrome type 1 (MIM#253310). Depletion of the *GLE1* gene in zebrafish models has shown apoptosis of neural precursors leading to a reduction in motor neurons and aberrant branching of motor axons [10]. A similar mechanism can be postulated in *THOC2* gene defects. 

The limitation of our study is the lack of functional evidence such as ribonucleic acid (RNA) or protein studies to decipher how the novel pathogenic variant causes the disease. However, the present *THOC2* gene pathogenic variant affecting the consensus splice site was considered to be pathogenic as per the loss of function mechanism as discussed. The present pathogenic variant is much proximal (intron 22) to all previously described splice sites or truncating variants (intron 28, intron 34, and deletion of exons 34 and 35) and hence, is a more severe type probably leading to a much smaller protein or no protein at all. We, therefore, postulate that amorphic or severe null pathogenic variants (possible complete loss of function) in the *THOC2* gene will lead to AMC phenotype rather than XLMR which are caused by hypomorphic/less severe (partial loss of function) pathogenic variants.

## Conclusions

The *THOC2* gene mutation can lead to recurrent fetal AMC, which is a severe presentation due to a novel mutation at consensus acceptor splice site at intron 22 and exon 23 junction. The inheritance is X-linked recessive. The mechanism of the splicing mutation is likely to be “amorphic” or severe loss of function type. This finding thus aids in the expansion of the clinical spectrum of *THOC2* gene-related disorder thereby aiding genetic counseling and prevention of further recurrences in such families. 
